# The Source of Palm Orientation Errors in the Signing of Children with ASD: Imitative, Motoric, or Both?

**DOI:** 10.3390/brainsci10050268

**Published:** 2020-04-30

**Authors:** Aaron Shield, Megan Igel, Kristina Randall, Richard P. Meier

**Affiliations:** 1 Department of Speech Pathology & Audiology, Miami University, Oxford, OH 45056, USA; igelme@miamioh.edu (M.I.); randalk4@miamioh.edu (K.R.); 2 Department of Linguistics, University of Texas at Austin, Austin, TX 78712, USA; rmeier@austin.utexas.edu

**Keywords:** autism spectrum disorder, sign language, imitation, cognition, language acquisition

## Abstract

Palm orientation reversal errors (e.g., producing the ‘bye-bye’ gesture with palm facing inward rather than outward as is customary in American culture) have been documented in the signing of deaf and hearing children with autism spectrum disorder (ASD) and in the imitation of gestures by signing and non-signing children with ASD. However the source of these unusual errors remains opaque. Given that children with ASD have documented difficulties with both imitation and motor skills, it is important to clarify the nature of these errors. Here we present a longitudinal case study of a single child with ASD, a hearing, signing child of Deaf parents. Samples of the child’s signing were analyzed at ages 4;11, 6;2, 10;2, and 14;11. Lexical signs and fingerspelled letters were coded for the four parameters of sign articulation (handshape, location, movement, and palm orientation). Errors decreased for handshape, location, and movement after age 4;11, but increased on palm orientation from 4;11 and remained high, exceeding 55% of signs by 14;11. Fingerspelled letters contained a large proportion of 180-degree reversals, which suggest an origin in imitation differences, as well as midline-facing errors, suggestive of a motor origin. These longitudinal data suggest that palm orientation errors could be rooted in both imitation differences and motoric difficulties.

## 1. Introduction

We previously presented the first report [1] on an aspect of the language development of native-exposed signing children with autism spectrum disorder (ASD). In that paper, we showed that three young children with ASD who had been exposed to American Sign Language (ASL) from birth by their deaf parents exhibited an unusual formational pattern in their signing: the reversal of the palm orientation parameter, such that signs normally produced with an outward-facing palm were produced with an inward-facing palm, or vice versa. Since such errors are not known to occur in the typical development of ASL beyond an early age, we speculated that such reversals could be unique to signing children with ASD and as such might be included in clinical criteria adapted for sign-exposed children. Interestingly, to-date signing children with ASD have not been found to produce pronoun reversals [2] like those characteristically found in the speech of some hearing children with ASD [3,4,5] as well as very young typically-developing hearing children [6,7,8], raising the possibility that the documented palm reversals could be a sign language analog to pronoun reversals in speech—that is, errors that occur due to the child’s difficulty understanding how linguistic forms shift between speakers/signers.

These palm reversal errors have thus provided an opportunity to speculate about the kinds of cognitive, linguistic, or motoric differences that might underlie their production by signers with ASD. At the time of our initial report, we followed the interpretation used in a review of 21 studies of imitation by children with ASD [9] which described difficulties with “self-other mapping”, the translation of others’ movements onto one’s own body. Particularly strong evidence of this interpretation came from a number of studies [10,11,12,13] which had found reversal errors in gesture imitation by hearing children with ASD that were of the same type as those we later documented in signing children with ASD. Although the errors we previously reported [1] were not errors of imitation, but rather of sign production, both elicited and spontaneous, we found it plausible that differences in imitation style could contribute to erroneous phonological representations of signs, thus accounting for the reversed palm orientation parameter in sign language production. We later elaborated on this hypothesis [14], describing a “visual matching strategy” in imitation that is characteristic of some learners with ASD, in which signs are imitated as they appear from one’s own perspective, resulting in palm orientation reversals and other erroneous sign productions, such as reversals of the direction of movement.

Despite the reasonable conjecture that such errors could be the result of an imitation difference, motor issues cannot be excluded as a possible cause of palm orientation errors. From 50 to 80% of children with ASD exhibit motor impairments [15,16,17,18], including basic motor skill deficits in reaching and walking [19,20], gross and fine motor incoordination [15,17,21], as well as deficits in praxis/motor planning [22,23,24,25,26], and such deficits have been found to extend to deaf, signing children with ASD [27]. Children with motor issues with the articulation of signs might execute signs with the palm facing the midline of the body, which is the default resting position of the palm when the arms are hanging at one’s sides. Producing inward- or outward-facing palm orientations requires the supination and pronation of the forearm, respectively. The ability to pronate and supinate the forearm develops throughout early childhood, with about 90% of typical children reaching mastery by age 6.5 [28]. Signers with motor disorders resulting from Parkinson’s Disease have been shown to neutralize the palm orientation parameter by producing signs toward the midline rather than with inward or outward orientation as a result of reduced motoric effort [29].

Given that children with ASD have documented difficulties with both imitation and motor skills, it is important to clarify the nature of the unusual sign articulation errors that we have documented in signing children with ASD. In particular, longitudinal data on the developmental trajectory of such errors in comparison with the other articulatory parameters of sign could be illuminating. In this regard it is possible to make predictions about what the developmental trajectory of articulatory parameters would look like under two hypotheses:

*Motor origin hypothesis*: Motor difficulties are predicted to result in the palm facing the midline (default resting position) rather than outward or inward (pronated or supinated). Furthermore, if motor issues are the sole or primary cause of palm orientation errors, then the error rate in palm orientation is predicted to: (a) mirror that of the other sign language parameters (handshape, location, and movement) and (b) decrease over time as motor skills improve.

**Figure 1:**
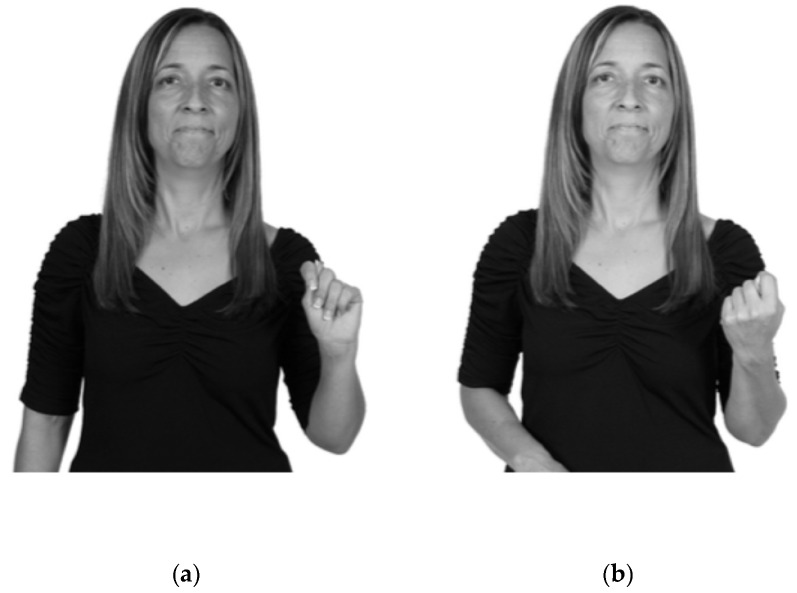
(**a**) Example of how the fingerspelled letter t is typically produced; and (**b**) How the fingerspelled letter t would be imitated with 180-degree reversal.

*Imitation hypothesis*: Differences in imitation are predicted to result in 180-degree reversal errors (signs specified for outward orientation produced with inward-facing orientation and vice versa); see Figure 1. Furthermore, if differences in imitation are the sole or primary cause of palm orientation errors, then the error rate in palm orientation is predicted to: (a) diverge from that of the other articulatory parameters (which are less affected by the visual matching imitation style), and (b) could remain relatively stable over time, as imitations solidify into mental (phonological) representations. 

A large number of the palm orientation reversal errors documented in our prior report [1] were produced on fingerspelled letters rather than on lexical signs: we reported 50 reversal errors on fingerspelled letters and five reversal errors on lexical signs. Fingerspelling is a system whereby the written alphabet of a spoken language is represented by different hand configurations. The fingerspelling system in ASL is one-handed; that is, each letter of the written alphabet is represented by a unique hand configuration (see Appendix A). Signed languages differ from each other in how they represent written alphabets as well as in the extent to which fingerspelling plays a role in the larger signed language. It is conventionally understood that the American Deaf community employs fingerspelling to a greater extent than in most other Deaf communities around the world [30]. 

Fingerspelling is most often used for proper names or for technical or novel terms for which a conventional lexical sign is lacking. Unlike lexical signs, which only employ one or two different handshapes [30], fingerspelling requires the signer to execute a series of different handshapes, one for each letter of the word being spelled. Lexical signs can be specified for different locations from the head to the waist or can be made in neutral space (e.g., the sign mother on the chin, the sign father on the forehead), [Links to video examples from the SignBank database [31] are provided for all lexical signs in the online version of the paper.] In contrast, fingerspelling in ASL is performed in a relatively small neutral space in front of the signer’s torso. Most fingerspelled letters are static handshapes without movement, with the exceptions of the letters j and z (Appendix A), while lexical signs draw from an extensive set of possible movements. Finally, while palm orientations of lexical signs can be specified to face up, down, toward the midline, to the sides, or toward or away from the signer’s body, fingerspelled letters in ASL all face outward from the signer’s body with a pronated forearm, with the exception of the letters g, h, p, and q. The letters g and h face inward, with the forearm rotated inward (supinated), while the letters p and q face downward, with a flexed wrist and pronated forearm (Appendix A), though note that there is a variant production of p with only very slight flexion of the wrist and supination of the forearm, resulting in inward palm orientation [32], but the participant in this study did not produce any tokens of this variant. 

In the sections that follow we distinguish between lexical signs and fingerspelled letters and analyze them separately. We do so for the following reasons: (1) we observed a large number of fingerspelling errors in our previous work [1]; and fingerspelled words (2) require the execution of a series of hand configurations in sequence; (3) are uncomplicated by changes in location; (4) are largely uncomplicated by changes in movement; and (5) present frequent opportunities for 180-degree palm reversals given their specification for outward-facing palm orientations.

This study presents a longitudinal case study of a single native signer with ASD, a hearing son of two Deaf parents, and analyzes the four articulatory parameters of his signs over a 10-year period, in order to shed light on the nature of palm orientation errors in ASD.

## 2. Materials and Methods

The parents of the participant gave their informed consent before including their child in the study. The study was conducted in accordance with the Declaration of Helsinki, and procedures were prospectively approved by the Institutional Review Board of the University of Texas at Austin (at ages 4;11 and 6;6; Protocol #2007-08-0022), Boston University (age 10;2; Protocol 2471E) and Miami University (age 14;11; Protocol 01375).

### 2.1. Participant

The child described here was one of the three natively sign-exposed children with ASD described previously [1]; in that work he was referred to as “Child 3”. He is a left-handed hearing male, diagnosed with ASD at age 2;6 by a licensed clinical psychologist. He has two Deaf parents who communicate primarily through ASL and a younger hearing brother. His parents indicated that he has received occupational therapy for low muscle tone affecting his fine motor skills. (While handedness is certainly a relevant factor in considering how children might imitate signs [14,33], the palm orientation parameter is unaffected by handedness. Therefore the child’s left-handedness is not considered further in our analyses.)

In addition to the data collected at age 6;6 reported previously [1], we visited the child at three different times over the course of ten years: at ages 4;11, 10;2, and 14;11. Over the course of those ten years we collected a number of standardized measures of language (both English and ASL), nonverbal intelligence, and ASD; these are reported below. He exhibits moderate ASD symptoms, by behavioral observation (ADOS-2) and by parent report (SCQ and AQ-Adolescent). He scores in the impaired range on nonverbal intelligence (TONI-4) and on receptive language for English (CELF-5; PPVT-4) and ASL (ASL RST).

#### 2.1.1. Autism

The Autism Diagnostic Observational Schedule, Second Edition (ADOS-2; [34]) was administered at age 9;11 by a clinician who had attained research reliability on the instrument and was fluent in English and ASL. The child’s total score of 15 (corresponding to a severity score of 6 on a scale of 1–10) indicated moderate ASD symptoms and was above threshold for autism classification. His mother completed the Social Communication Questionnaire (SCQ; [35]), Lifetime Form, at 10;2 and 14;11; his total score was above threshold for ASD risk at both ages (raw score of 14 at 10;2 and raw score of 16 at 14;11). Additionally, his mother completed the Autism Quotient (AQ; [36]), Adolescent Version, at 14;11. His score of 34 was above the threshold score for ASD of 32.

#### 2.1.2. Intelligence

We administered the Test of Nonverbal Intelligence, Fourth Edition (TONI-4; [37]) at age 10;2 and 14;11. At 10;2 he achieved a raw score of 6, which translates into a standard score of 69, just under 2 SD below the mean. At 14;11 he achieved a raw score of 25, corresponding to a standard score of 86, or 17th percentile for his age and an age equivalent of 9;0.

#### 2.1.3. Language

Our participant is the bimodal bilingual child of Deaf parents. It is important to note that there is no established profile for bimodal bilingual children exposed to both a signed language and a spoken language, such as the hearing children of Deaf adults [38]. However, hearing children of Deaf adults typically have speech that is equivalent to monolingual hearing children by about age 7 [39]. At age 6;6 our participant’s mother filled out the Language Proficiency Profile, Second Edition (LPP-2; [40]), a parent report measure to estimate global communication skills. His total score of 26 indicated language well below his chronological age. At age 10;2 we administered both the Peabody Picture Vocabulary Test, Fourth Edition (PPVT-4; [41]) and the American Sign Language Receptive Skills Test (ASL RST; [42]) to obtain measures of his receptive skills in English and ASL. He obtained a standard score of 46 on the PPVT-4 (1st percentile), corresponding to an age equivalent of 4;1. On the ASL RST, he obtained a raw score of 6, corresponding to an age equivalent of under age 3, the youngest age for which norms on this test are given. At 14;11 we repeated the ASL RST and added the Receptive Language Index subtests of the Clinical Evaluation of Language Fundamentals, Fifth Edition (CELF-5; [43]). On the ASL RST he achieved a raw score of 12, corresponding to a standard score of 71, about 2 SD below the mean. On the CELF-5, he achieved a standard score of 45, more than 2 SD below the mean.

#### 2.1.4. Prior report

In our previous report [1], we described data collected from the child when he was age 6;6. At 6;6, the child produced 59 signs, of which 35 (59.3%) contained one or more articulatory errors. For a summary of the child’s articulation errors at that age, see Table 1.

### 2.2. Procedure

The child was observed at home at all four time points. At age 4;11, he was observed in an unstructured, naturalistic interaction with his Deaf father, who engaged with him while reading to him from a picture book. At 6;6 and 10;2, he was observed interacting with the first author, a hearing researcher fluent in ASL, who performed a series of experimental tasks, including eliciting fingerspelled words and lexical signs. At 14;11, he was observed interacting with his Deaf mother, who asked him a series of questions in ASL about friends, school, books, and movies.

### 2.3. Coding

Using ELAN (EUDICO Linguistic Annotator) multimodal coding software [44], we coded 12 continuous minutes from each time point (ages 4;11, 6;6, 10;2, and 14;11) for all signs produced. For age 6;6, previously reported [1], we coded a new 12-minute span of video. Each letter of a fingerspelled word was coded and counted as an individual sign. Each sign was coded for handshape, location, movement, and palm orientation (inward, outward, upward, downward, or midline-facing). The coded value for each parameter was scored as being produced correctly or as an error based on standard citation forms; we used the ASL Signbank as a reference (see https://aslsignbank.haskins.yale.edu/) [31]. Where movement segments were deleted, resulting in a missing second location, errors were coded as movement errors only. Errors were qualitatively described so as to allow for further analysis.

### 2.4. Reliability

To ensure the reliability of the coding system, two 5-minute segments (one from age 4;11 and one from 14;11) were blindly recoded by a second trained coder experienced in the coding of ASL. Differences in coding were discussed by both coders and disagreements were resolved through consensus. The main coder then adjusted the rest of the coding to reflect the decisions made through consensus discussion with the second coder.

## 3. Results

Table 2 presents a comparison of the overall number of signs produced and the number of signs produced per minute. Overall sign production increased over time, although note that we have counted individual fingerspelled letters as separate signs. Importantly, the child’s fingerspelling increased over time, which could account for the greater number of signs produced, especially at 14;11. This increase in fingerspelling is in line with other reports of the developmental trajectory of fingerspelling, which have shown that fingerspelling to children by adults increases as the children mature, and the fingerspelling produced by such children increases in turn [30].

Table 3 presents the total number of lexical signs and fingerspelled letters produced at each time point, and the number of errors on each of the four sign parameters produced for both types of signs. Note that fingerspelled letters are all produced in neutral space and generally do not exhibit movements, except for the letters j and z (see Appendix A); thus location and movement errors are unlikely on fingerspelled letters.

**Figure 2:**
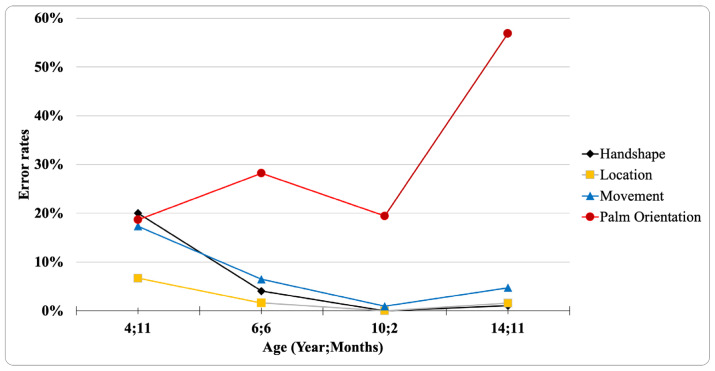
Proportion of signs exhibiting errors in the four sign parameters at four ages.

Figure 2 shows the child’s error rates on the four sign articulation parameters across the four time points, collapsing lexical signs and fingerspelled letters. Error rates for handshape, location, and movement decrease over time, while the palm orientation parameter shows the opposite trend, increasing to an error rate of over 50% at age 14;11. By comparison, studies of the early acquisition of phonological parameters in ASL have found that the handshape parameter is the most error-prone early in development, while location is acquired earliest, as appears to be the case for this participant at age 4;11. Most studies of phonological development in ASL have primarily focused on children who are much younger than the participant in this study, i.e., under age 2 [45,46,47].

We distinguished three different types of palm orientation errors: 180-degree reversals (substitutions of inward palm orientation for outward or vice versa), midline errors (neutralization of the palm orientation parameter such that the palm faced toward the midline rather than inward, outward, up, or down), and other errors (e.g., substitutions of an upward- or downward-facing palm for inward or outward). Table 4 reports the frequency of each error type of error at each age.

Looking across all of the palm orientation errors produced in our sample, 159 of 182 errors (87.3%) were produced on fingerspelled letters while the remaining 23 errors (12.6%) were produced on lexical signs. We report all fingerspelled letters in Table 5 below. Note that a number of fingerspelled names produced at age 14;11 were redacted to protect the participant’s identity. In these redacted fingerspelled names, the participant produced 7 names: four 5-letter names and one 4-letter name with all letters produced facing the midline; one 4-letter name with all letters reversed, and one 4-letter name with the first three letters reversed and the last letter produced with correct outward orientation. There were no instances of g, h, p, or q in these names, so the target orientation for all letters was outward.

It is clear from Table 5 that the child produced fingerspelled letters with inconsistent palm orientation. Indeed, he produced certain fingerspelled handshapes with different palm orientations during the same session (e.g., with in/mid/outward palm orientation on e and o at 6;6 and d, a, and y at 14;11) and varied the palm orientation of fingerspelled letters at different ages (e.g., l outward at 10;2 but inward and midline-facing at 14;11).

**Figure 3:**
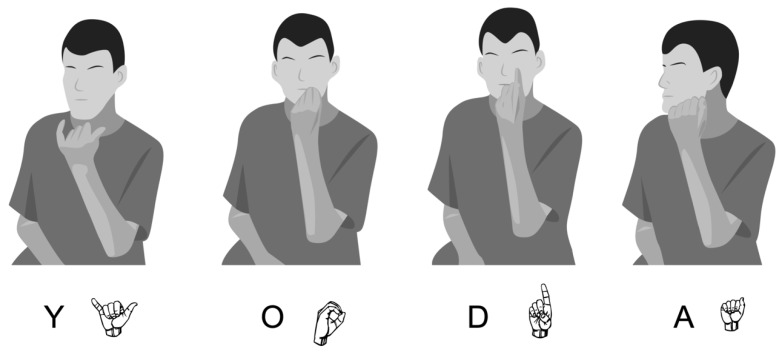
The fingerspelled word #yoda produced with reversed, inward palm orientation on each handshape at 14;11. The word was produced rapidly and fluently, unlike the labored production of #father in Figure 4.

**Figure 4:**
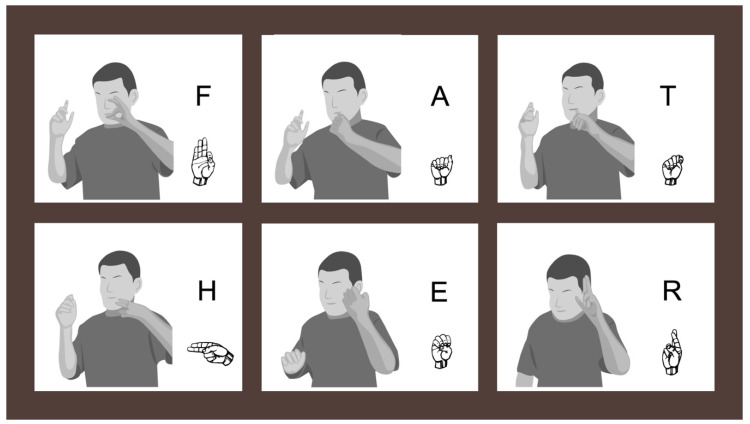
The fingerspelled word #father produced on the left hand at 10;2 with correct outward palm orientation on the letter f, mid-facing orientation on the letter a, correct outward palm orientation on the letter t, correct inward orientation on the letter h, incorrect reversed palm orientation on the letter e, and correct outward palm orientation on the letter r. Note the lack of inhibition of movement of the non-signing right hand, indicative of a lack of motor control.

In order to understand the inconsistency exhibited in palm orientation, we examined how palm orientation errors occurred within fingerspelled words. First, some words maintained a consistent palm orientation, be it correct (outward) as in #door. at 6;6, or incorrect such as the midline orientation exhibited in #swing at 14;11 or reversed (inward) as in the #yoda example illustrated in Figure 3. [As is conventional in the literature, fingerspelled words are denoted by a preceding pound sign (#)]. However, we also found instances in which the child switched between (correct) outward palm orientation and (incorrect) inward palm orientation within the same fingerspelled word. Words that follow this pattern of inconsistency include #teach, #phone, #mother, and #father (Figure 4) at 10;2. Recall that all fingerspelled letters are typically produced with the palm facing outwards (with pronated forearm) except for g and h (which face inward with supinated forearm), and p and q (which face downward, with pronated forearm and flexed wrist). This difference in specification for palm orientation means that in words that contain these four letters, the signer must switch between outward, inward, and downward palm orientations in the course of normal signing, which requires the pronation, supination and re-pronation of the forearm (as well as wrist flexion for p and q). The child in this study also produced words without errors in which he successfully switched between inward, outward, and downward palm orientations, such as the word #telephone at 6;6, where p and h were produced with correct downward and inward orientations, respectively, and all other letters with correct outward orientation (though note the substitution of i for l). Other examples of this include the words #school (produced without the c), #girl, #chair, and #bug produced at 10;2. However there are also examples in which the child produced a reversed palm orientation on letters that are adjacent to h. Examples that follow this pattern include #chair at 6;6, #teach, #phone, #mother, and #father (Figure 4) at 10;2, and #school and #theincredibles at 14;11.

## 4. Discussion

We have documented the development of the four parameters of sign articulation over a period of ten years in a single child with ASD, a natively sign-exposed hearing child of two Deaf parents. This is the first time that the sign development of a native signer with ASD has been studied longitudinally. We had hypothesized that the palm orientation errors documented previously [1] could have imitative or motoric origins, and that the developmental trajectory of the palm orientation parameter, in comparison with the other parameters of sign language development, could shed light on this question. Here we evaluate the evidence for both hypotheses.

Is there evidence in favor of the motor origin hypothesis? Yes. The strongest evidence is the occurrence of palm orientation errors produced toward the midline rather than inward or outward. Such errors accounted for 47.8% of the palm orientation errors in our sample, occurred at all ages studied, and reflect the neutralization of the palm orientation parameter toward a default resting position [29]. The fact that errors on the three other parameters (handshape, location, and movement) decrease over time suggests a developmental trajectory of improvement in motor skills that does not extend to palm orientation; in particular, the handshape parameter, which has the highest error rate at age 4;11, decreases nearly to zero by 10;2, and remains stable at 14;11. Numerous studies have found that, of the three major parameters of handshape, location, and movement, handshape is the parameter that is mastered latest in typical development [45,46,48,49,50,51,52,53], probably due to the late development of the fine motor control required to produce handshapes accurately (though note that not all of these studies examined palm orientation as a separate parameter).

A second source of evidence in favor of the motor hypothesis is the fact that the child sometimes reversed palm orientation on letters that were adjacent to the inward-facing letters g and h. This suggests that the child anticipated the switch in palm orientation on a subsequent letter (as in the c in #chair at 6;6 or the t in #mother at 10;2), or failed to reorient his palm to face outward (i.e., re-pronate the forearm) following one of the inward facing letters (as in the o in #phone, the e in #father, and the e in #mother at 10;2). Additional evidence of motor control issues include the lack of inhibition of the non-dominant hand shown in Figure 4, and the unusually high signing shown in Figure 3 and Figure 4.

Given the evidence for motor impairment causing palm orientation errors, is there also evidence in favor of the imitation hypothesis? Here, too, the answer appears to be yes. Unlike the other parameters of sign formation (handshape, location, and movement), which show a clear decrease in error rate over the ten-year period, palm orientation errors increase over time, to above 50% at age 14;11, and reversal errors made up nearly half of all palm orientation errors documented in this study (82 out of 182 errors; 45.1%). Particularly striking are fingerspelled words that do not include the letters g and h but which were produced with consistently inward palm orientation, as in #park, #paris, and #yoda (Figure 3) at 14;11. It is unlikely that such 180-degree reversal errors would result from motoric difficulties, since the supination of the forearm entailed in the production of inward palm orientations is as motorically difficult to execute as the pronation of the forearm entailed in outward palm orientations. Instead, these reversal errors are suggestive of differences in imitation during the sign learning process in which the child reproduces what he sees from his perspective (“visual matching”), yielding forms with reversed palm orientation. It is worth noting that most of the 180-degree reversal errors described here involve the substitution of an inward-facing palm orientation (supination) for an outward-facing palm orientation (pronation); 75 of the 82 (91.5%) 180-degree reversal errors described here fall into this category. We believe that this finding is again due to the fact that nearly all fingerspelled letters are typically produced with an outward-facing palm, and the child reported on here tended to reverse palm orientation on fingerspelled letters. Despite this trend, a minority of errors (7 of 82 or 8.5%) involved the substitution of an outward-facing palm for an inward-facing palm, showing that reversal errors can replace inward with outward palm orientations as well as outward with inward palm orientations. Other errors of this type, such as the production of the lexical sign butterfly with outward-facing rather than inward-facing palms, have been reported before [33]. More study is warranted to better understand which lexical signs could be subject to palm reversals of this type.

Why should palm orientation errors increase over time? For this question, too, there appears to be a clear answer: palm orientation errors surface most often in fingerspelling, and fingerspelling increases with age, as children become more literate and incorporate more English words into their vocabulary [30]. Indeed, fingerspelling accounted for 110 of the 112 (98.2%) palm orientation errors produced by this child at 14;11. Fingerspelled letters require the pronation of the forearm such that the signer’s palm faces outward on all letters except for g and h (produced with supination such that the palm faces inward) and p and q (produced with pronation and wrist flexion such that the palm faces downward). The tendency to reverse palm orientation on fingerspelled letters was previously observed for a different child at age 7;5 [1], who produced 61 palm orientation errors, 50 of which were fingerspelled letters produced with inward palm orientation rather than outward. The other 11 errors were midline errors, confirming the patterns observed here: reversals and midline substitutions on fingerspelled letters. Similar to the case discussed here, this child produced a low rate of errors on the other sign parameters (6 movement errors, 1 handshape error, and 0 location errors out of 94 sign tokens), suggesting overall good motor control.

It is important to note that fingerspelling is typically directed toward an interlocutor. In this sense, palm orientation in fingerspelling could also reflect pragmatic competence: the signer must understand that their signing should be produced facing in the direction of their interlocutor. Typically-developing signing children do not produce reversal errors of this type on fingerspelled words; in our previous work no such errors were produced by a sample of 12 deaf children of deaf parents between age 3;7 and 6;9 [1], and to our knowledge there are no other instances of such errors in the literature. The idea that difficulties with pragmatics could underlie the palm reversals documented in the signing of children with ASD suggests a parallel between these errors and the pronoun reversals documented in the speech of hearing children with ASD, as the latter errors have been interpreted as evidence of challenges with understanding how discourse roles shift between interlocutors during conversation [6,54,55].

Despite the frequency with which palm orientation reversal errors occurred on fingerspelled letters, palm orientation reversals also occur somewhat infrequently on other types of signs, such as lexical signs. In our previous work [1], we noted the reversal of palm orientation from inward to outward on the lexical sign flashing-light, and outward to inward on the sign bye-bye, both produced by the child described here. In that work as well as in this study, we also find evidence of reversed palm orientation on number signs (which in ASL are formationally similar to fingerspelling), though these errors should be interpreted with caution since there is variability in the production of these signs depending on whether numbers are ordinal, cardinal, or part of a series such as a postal code [56].

One puzzling result is that the palm orientation value for individual signs was variable and unstable. That is, the same sign—especially the same fingerspelled letter—was produced with up to three different palm orientations, and this variability occurred both within the same session as well as across different sessions. If the child had a fixed mental representation of the palm orientation parameter for a given sign, then we would expect him to produce the sign with the same palm orientation value every time he produced that sign. We had hypothesized that differences in imitation style (such as the visual matching strategy, in which the child reproduces signs exactly as they appear from his perspective) could result in mental representations with incorrect palm orientation values [14]. Instead of a fixed but erroneous representation of palm orientation, we propose that the variability of input results in an *unstable* or *underspecified* mental representation of the palm orientation parameter. Children exposed to signs observe signs produced from various angles: whether facing the adult signer head-on, or from the side, or from behind a parent as that adult signs to others, or from every conceivable angle in between. This variability in sign input could result in an unstable value for the palm orientation parameter in the child’s mental representation of the sign, or indeed no palm orientation value at all. As it happens, the palm orientation parameter may carry a low functional load compared to the other parameters that signs are composed of. Minimal pairs for palm orientation are signs which differ only in their palm orientation value, proving the phonological contrastiveness of the palm orientation parameter; e.g., children versus things [57]. Although minimal pairs for palm orientation do exist in ASL, they appear to be rare, especially compared to minimal pairs for location, handshape, and movement. 

We must caution that these results may not be reflective of all signers with ASD. Indeed, ASD is characterized by its diversity of presentation, and this is true both in terms of language ability and motor skills. However, it appears clear that differences in imitation lead both hearing and deaf children with ASD to imitate gestures and produce signs in ways that are unlike typical children, and this paper argues that although motor difficulties appear to be an important factor in the production of palm orientation errors, motor impairment alone cannot account for all of the errors observed. We would not predict or expect, however, that all signing children with ASD would produce palm orientation reversals. Indeed, such reversals may occur within a subset of children whose language or ASD severity fits a specific cognitive profile, though such a profile has not yet been identified. It is worth noting that the child described here has significant intellectual disability. In contrast, the two children described in our previous report who produced palm orientation reversals did not exhibit intellectual disability [1]; both children were in the average range of intelligence but in the below-average range of language ability. It thus appears plausible that palm orientation reversal errors are unrelated to overall intelligence but could be linked to lower language abilities. A related issue is whether the differences observed could manifest in linguistic structures other than the phonological form of the sign (e.g., role-shift requiring the assumption of different perspectives, or various types of path movements entailed in agreement verbs). We have not observed either of these phenomena due to the overall low level of expressive sign language exhibited by this participant, but future research should investigate whether signing children with ASD experience difficulty with other aspects of the linguistic system that are rooted in motoric, imitative, or other cognitive challenges or differences.

Although the quantity of signs produced in the 12-minute segments increased over time (from 6.33 signs/minute to 16.42 signs/min), the differences in procedures at each time point do not permit direct comparisons. In particular, the increased fingerspelling produced at age 14;11 was largely responsible for the increase in total number of signs produced at the later age. Gains were also observed on the only language measure that was administered at two time points, the ASL RST (at ages 10;2 and 14;11), on which the child increased his raw score from 3 (age equivalent < 3 years) to 12 (age equivalent of approximately 4.5 years). Thus the increase in palm orientation errors occurred *despite* evidence of gains in both receptive and expressive language.

As palm orientation reversals have been documented in a variety of contexts (spontaneous signing, elicited signing, and in the imitation of gestures) and in a variety of populations of children with ASD (hearing children, deaf children, non-signers, and signers), we suggest that such reversals be considered a red flag for ASD diagnosis if they occur past the early developmental period. In particular, if diagnostic and screening instruments are adapted for signing children, we believe that it would be important to include items probing whether or not children produce palm reversals, as such errors rarely occur in typical development beyond the first two years of age.

## 5. Conclusions

This study represents the first longitudinal study of a signer with ASD. It demonstrates that palm orientation errors (both 180-degree palm reversals as well as midline-facing errors) can persist beyond childhood and into adolescence. Such errors are notable for both clinical and theoretical reasons: they could serve as a modality-specific marker of ASD, and as such could be incorporated into adapted diagnostic and screening instruments for signing children, and they also provide insight into the mechanisms (both imitative and motoric) that lead to such errors. Future studies are needed to help clarify how frequently such errors occur in the population of signing children with ASD, and whether there is a specific cognitive profile of children who produce them. These studies will be crucial for a better understanding of why some children with ASD produce these unique errors, and what kinds of differences could lead to their production.

## Figures and Tables

**Table 1:** Articulation errors previously reported at age 6;6.

Parameter	Number of Errors	Description of Errors
Location	3	On the sign orange, he failed to raise his hand from the resting position in his lap and therefore made the sign in contact with his knee rather than his chin (confirmed by maternal repetition immediately afterward) and produced the sign ice-cream in neutral space rather than at the chin. Finally, he produced the sign star without contact between the hands, at chest level rather than chin/head level
Handshape	9	He produced a 4-handshape instead of an h-dot handshape (i.e., a handshape with the first and second fingers extended and together, third and fourth fingers closed, and thumb extended) on rabbit, a 5-handshape instead of a v-handshape on dance (4 tokens), a baby-c-handshape instead of a g-handshape on the sign green, an a-handshape instead of an x-handshape on the sign apple, an 8-handshape instead of a g-handshape on the sign chicken, and a 5-handshape instead of an h-dot handshape on the sign horse
Movement	23	Forward movement (outward) rather than inward on the sign lion (two tokens) and computer (one token). He did not execute a path movement on several signs that normally exhibit path movement (such as elephant and giraffe), and reduced movement on several signs that typically exhibit repeated cycles of movement (e.g., horse, duck, monkey, bear and chicken). He deleted the movement segment entirely on the sign ice-cream. Other simplifications included the loss of the non-dominant hand on the sign dance as well as the dropping of one hand from a two-handed sign (bear, monkey). Several signs exhibited wild, uncontrolled movement, which were only interpretable because the parent repeated the sign with the correct form; these included dance and blue. Finally, he produced the sign yes with a forearm rotation rather than with a nodding movement of the wrist (two tokens)
Palm orientation	4	Two substitutions of an inward orientation for an outward orientation (on the sign flashing-light as well as a wave gesture) and the substitution of a downward orientation for a midline-facing orientation (turtle) and for an inward orientation (three)

**Table 2:** Comparison of quantity of signs produced and error rates across time points

Age	4;11	6;6	10;2	14;11
Number of sign tokens produced	76	124	108	197
Sign tokens/min	6.33	10.33	9.0	16.42

**Table 3:** Errors on lexical signs and fingerspelled letters at each age, classified by parameter.

AGE	4;11		6;6		10;2		14;11	
	Lexical (*N* = 72)	Fingerspelling (*N* = 4)	Lexical (*N* = 69)	Fingerspelling (*N* = 55)	Lexical (*N* = 43)	Fingerspelling (*N* = 65)	Lexical (*N*= 76)	Fingerspelling (*N* = 121)
Handshape error	15 (20.8%)	0	5 (7.2%)	0	0	0	2 (2.6%)	0
Location error	5 (6.9%)	0	2 (2.9%)	0	0	0	3 (3.9%)	0
Movement error	13 (18.1%)	0	8 (11.6%)	0	1 (2.3%)	0	9 (11.8%)	0
Palm orientation error	14 19.4%)	0	5 (7.2%)	30 (54.5%)	2 (4.7%)	19 (29.2%)	2 (2.6%)	110 (90.9%)

**Table 4:** Palm orientation errors by type at each age.

Error Type	4;11	6;6	10;2	14;11	Total
180-degree reversal errors	1 (7.1%)	15 (42.9%)	8 (36.4%)	58 (50.9%)	**82 (45.1%)**
Midline errors	2 (14.3%)	18 (51.4%)	13 (59.1%)	54 (47.4%)	**87 (47.8%)**
Other errors	11 (78.6%)	2 (5.7%)	0	0	**13 (7.1%)**
**Total**	**14**	**35**	**21**	**112**	**182**

**Table 5:** Fingerspelled letters produced at each age. Letters produced with correct outward palm orientation (all letters except g, h) are represented in plain font, letters produced with correct inward palm orientation (g, h) are underlined, while letters that exhibited 180-degree reversals are bolded and letters produced with midline errors are italicized. Some tokens contain spelling errors produced by the child, e.g., the substitution of i for l.

	4;11	6;6	10;2	14;11
	R	W	B-A-L-L	**S**-*C*-H
	B	F	P-A-P-E-R	**S-C**-H-**O-O-L**
	D-W	V	G-I-R-L	*S-W-I-N-G*
		**B-O-O-K**	S-H-O-O-L	[redacted]
		D-O-O-R	B-I-R-D	[redacted]
		**C**-H-**A-I-R**	T-E-*A*-**C**-H	[redacted]
		*W-A-T-C-H*	**P**-P-**H**-**O-N-E**	Y
		*S-H-O-E-S*	*D-E*-S-K	[redacted]
		*T-A-B-I-E*	*C*-H-*A*-I-R	A
		*C-A-P*	*D-O-L-L*	*D-I-D*
		**B-E-D**	F-*A*-T-H-E-R	*B-E-D-A*
		S-C-I-S-S-O-R-S	M-*O*-**T**-H-**E**-R	[redacted]
		T-E-I-E-P-H-O-N-E	W-V-A-N	[redacted]
		**K**	B-U-G	*B-E*
				**D-L**-F-**W**
				M-D
				*N-A-D-D-W*
				M-D
				**P-A-R-K**
				**P-A-R-I-S**
				C
				[redacted]
				**D-A**-*S*-**R**
				**D-A**
				**T**-H-**E-I-N-C-R-E-D-I-B-L-E-S**
				**A-R-L**
				**P-E-T-E-R**-R
				R
				**R**
				**D**
				**Y-O-D-A**
Total number of fingerspelled letters produced	4	55	65	121
Total number of midline errors	0	18 (32.7%)	11 (16.9%)	53 (43.8%)
Total number of reversed letters	0	12 (21.8%)	8 (12.3%)	57 (47.1%)

**Figure A1:**
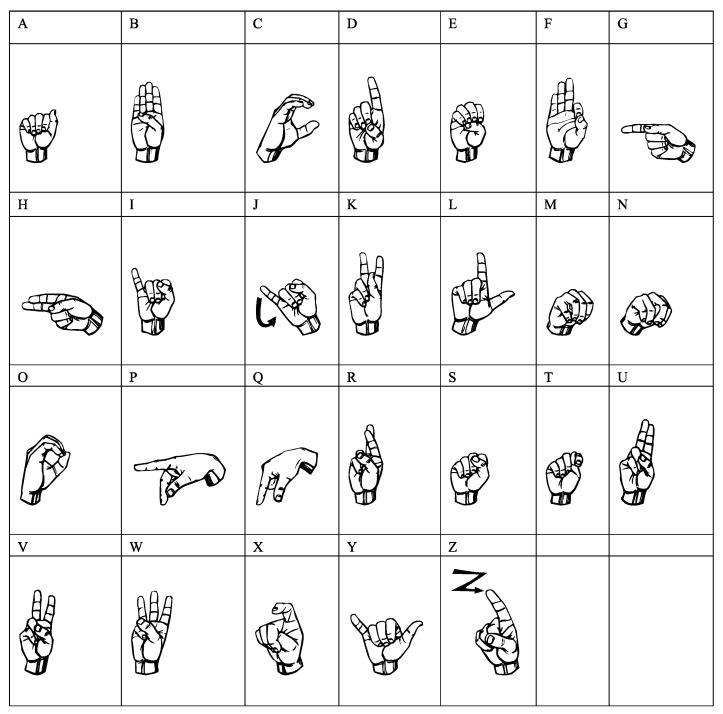
ASL Fingerspelling Handshapes.
